# Comparative analysis of de novo genomes reveals dynamic intra-species divergence of NLRs in pepper

**DOI:** 10.1186/s12870-021-03057-8

**Published:** 2021-05-31

**Authors:** Myung-Shin Kim, Geun Young Chae, Soohyun Oh, Jihyun Kim, Hyunggon Mang, Seungill Kim, Doil Choi

**Affiliations:** 1 Plant Immunity Research Center, Plant Genomics and Breeding Institute, Department of Agriculture, Forestry and Bioresources, College of Agriculture and Life Sciences, Seoul National University, Seoul, 08826 Korea; 2 Interdisciplinary Program in Agricultural Genomics, Seoul National University, Seoul, 08826 Korea; 3 Department of Environmental Horticulture, University of Seoul, Seoul, 02504 Korea

**Keywords:** Hot pepper, Genome, Disease resistance, NLR evolution, Copy number variation

## Abstract

**Background:**

Peppers (*Capsicum annuum* L.) containing distinct capsaicinoids are the most widely cultivated spices in the world. However, extreme genomic diversity among species represents an obstacle to breeding pepper.

**Results:**

Here, we report de novo genome assemblies of *Capsicum annuum* ‘Early Calwonder (non-pungent, ECW)’ and ‘Small Fruit (pungent, SF)’ along with their annotations. In total, we assembled 2.9 Gb of ECW and SF genome sequences, representing over 91% of the estimated genome sizes. Structural and functional annotation of the two pepper genomes generated about 35,000 protein-coding genes each, of which 93% were assigned putative functions. Comparison between newly and publicly available pepper gene annotations revealed both shared and specific gene content. In addition, a comprehensive analysis of nucleotide-binding and leucine-rich repeat (NLR) genes through whole-genome alignment identified five significant regions of NLR copy number variation (CNV). Detailed comparisons of those regions revealed that these CNVs were generated by intra-specific genomic variations that accelerated diversification of NLRs among peppers.

**Conclusions:**

Our analyses unveil an evolutionary mechanism responsible for generating CNVs of NLRs among pepper accessions, and provide novel genomic resources for functional genomics and molecular breeding of disease resistance in *Capsicum* species.

**Supplementary Information:**

The online version contains supplementary material available at 10.1186/s12870-021-03057-8.

## Background

Peppers (*Capsicum* spp.), which are among the most important vegetable crops and consist of about 35 species, produce beneficial molecules such as vitamin C, pigments, and capsaicinoids [1–4]. In 2018, global pepper production was ~ 60 million tons with a trade value of ~ 16 billion USD [5]. The most widely cultivated pepper species, *Capsicum annuum* (2n = 24) has a large genome with an estimated length above 3.0 Gb [2–4]. Currently, four assembled genomes are available for *C. annuum* [2–4, 6]. Genomic resources, including transcriptomes, variomes, and proteomes, have also accumulated in public databases [7, 8]. Nonetheless, more resources are needed to identify genomic and genetic features that provide insight into agronomic traits and phenotypic variations.

Advances in next-generation sequencing (NGS) and long-read sequencing technology have accelerated the sequencing and assembly of plant genomes. To date, hundreds of plant genomes have been published, and these sequences represent essential resources for breeding research [9, 10]. Specifically, population genetic studies have been conducted using those reference genomes, along with resequencing data, to identify genomic variations associated with important agronomic traits [11, 12]. However, due to extreme intra-genomic variations, one reference genome cannot represent the whole-gene repertoire of a plant species [9, 13]. To overcome such limitations, pan-genome projects have been conducted in major crops such as rice, maize, and tomato, with the goal of constructing integrated genome sequences that represent the whole-gene repertoires of the target species [14–16]. These pan-genome analyses using multiple genome resources could provide a platform for plant breeding-associated agronomic traits such as resistance to biotic and abiotic stresses [13].

Nucleotide-binding, leucine-rich repeat genes (NLRs) have been rapidly amplified and diversified in plants. The domain architecture of NLRs was classified into three main components: An N-terminal domain including a toll/interleukin-1 receptor homology, a coiled-coil, or a resistance to powdery mildew 8, central nucleotide-binding (NB-ARC) domain, and a C-terminal domain including leucine-rich repeat. In particular, the conserved NB-ARC domain is mostly used for defining NLRs [17]. Extensive intra- and inter-species comparisons have been performed on NLRs [18, 19]. For example, a species-wide study in 64 *Arabidopsis thaliana* accessions, termed the pan-NLRome, revealed the process of NLR evolution, including the diversification of NLR domain architectures and their specific selection patterns within the species [18]. Although pepper has a large expanded pool of NLRs [6], the complexity and variation of these genes within the species have not been previously examined.

Here, we report genome assemblies and annotations for two *C. annuum* accessions: the sweet bell pepper ‘Early Calwonder (ECW)’ and the hot chili pepper ‘Small Fruit (SF)’. Comparative analyses of newly assembled and publicly available pepper genomes revealed the evolutionary relationships and genomic variations among pepper accessions. We also re-annotated NLRs and constructed a physical NLR map, based on the reference pepper genome ‘Criollo de Morelos-334’ (CM334), with the two assembled genomes from this study (ECW and SF) and other publicly available *C. annuum* accessions (Zunla-1 and Chiltepin). We identified genomic regions in the NLR map where intra-specific repertoires of NLR genes exhibited significant copy number variations (CNVs) among pepper accessions. Extensive comparison of those regions indicated that CNVs of NLRs could have arisen by accumulation of intra-specific sequence mutations in pepper genomes. The newly constructed genome assemblies and annotations, along with the NLR map, provide a valuable resource for functional genomics and molecular breeding of disease resistance in *Capsicum* spp.

## Results

### Genome sequencing, assembly, and annotation

Two pepper genomes were assembled and annotated by the described pipeline (Supplementary Fig. 1). Using the Illumina HiSeq X-ten and NovaSeq 6000 platforms, we generated 460.2 Gb of raw sequence, representing 146.8 × and 145.8 × coverage of the ‘Early Calwonder (ECW)’ and ‘Small Fruit (SF)’ genomes, respectively (Supplementary Table 2). After removing unnecessary reads, genome sizes were estimated as 3.14 and 3.18 Gb for ECW and SF, respectively, based on the 19-mer frequency distribution (Supplementary Fig. 2 and Supplementary Table 3). A total of 83,882 and 87,732 (~ 2.84 Gb length each) initial contigs with N50 lengths of 114 and 110 kb were assembled into 44,107 and 44,731 scaffolds of 2.88 Gb length each covering 91.7% and 90.6% of the expected genome sizes for ECW and SF, respectively (Table 1). We detected 1,323 (96.2%) and 1,316 (95.7%) conserved single-copy orthologs in genome assemblies of ECW and SF using BUSCO [20], respectively, indicating equivalent assembly quality compared to other pepper genomes (Supplementary Table 4).

**Table 1:** Summary of genome assembly, gene annotation, and BUSCO validation

	ECW	SF	CM334^a^	Zunla-1^b^	Chiltepin^b^
Number of scaffolds	44,107	44,731	37,989	967,017	1,973,483
Total length of scaffolds	2.88 Gb	2.88 Gb	3.03 Gb	3.35 Gb	3.48 Gb
N50 of scaffolds	385 kb	418 kb	2.5 Mb	1.23 Mb	446 kb
Number of contigs	83,882	87,732	337,328	1,102,606	2,109,725
Total length of contigs	2.84 Gb	2.84 Gb	2.96 Gb	3.21 Gb	3.30 Gb
N50 of contigs	114 kb	110 kb	30 kb	55 kb	52 kb
Number of genes	35,355	35,158	35,884	35,336	34,476
Average/total CDS length	1,113 bp / 39 Mb	1,174 bp / 41 Mb	1,091 bp / 39 Mb	1,020 bp / 36 Mb	1,006 bp / 35 Mb
Average exon/intron length	240 bp / 874 bp	242 bp / 793 bp	243 bp / 945 bp	239 bp / 716 bp	249 bp / 734 bp
Proportion of complete BUSCOs	91.2%	92.3%	88.8%	90.6%	84.6%

^a^ The genome and annotation described in Kim et al. [3, 6]

^b^ The genome and annotation described in Qin et al. [4]

Gene annotation predicted 35,355 and 35,158 protein-coding genes in ECW and SF, respectively (Table 1). Of those, 32,983 (ECW, 93.3%) and 32,838 (SF, 93.4%) have been assigned putative functional descriptions in public databases (Table 1 and Supplementary Table 5). Comparison of the genes in the ECW and SF genomes with publicly available pepper gene annotations revealed that the lengths of annotated genes were similar among all pepper genomes (Supplementary Fig. 3). Validation of annotated genes using BUSCO detected 1,254 (91.2%) and 1,269 (92.3%) conserved single-copy orthologs in ECW and SF, respectively (Table 1 and Supplementary Table 4). Given the similar gene structure and validation of genome assemblies and annotated genes relative to publicly available pepper genomes, these results indicate that our assemblies and annotations of ECW and SF are reliable.

Repeat analysis revealed that 2.64 (ECW, 84.1%) and 2.63 Gb (SF, 82.7%) were annotated as repeat sequences, whereas only 1–2% of the assembled genomes were assigned as genes. LTR/Gypsy elements represented 68.8% of all annotated repeat types (Supplementary Table 6), consistent with the repeat contents of other pepper genomes [3].

### Clustering and phylogenetic analyses of annotated genes

**Fig. 1:**
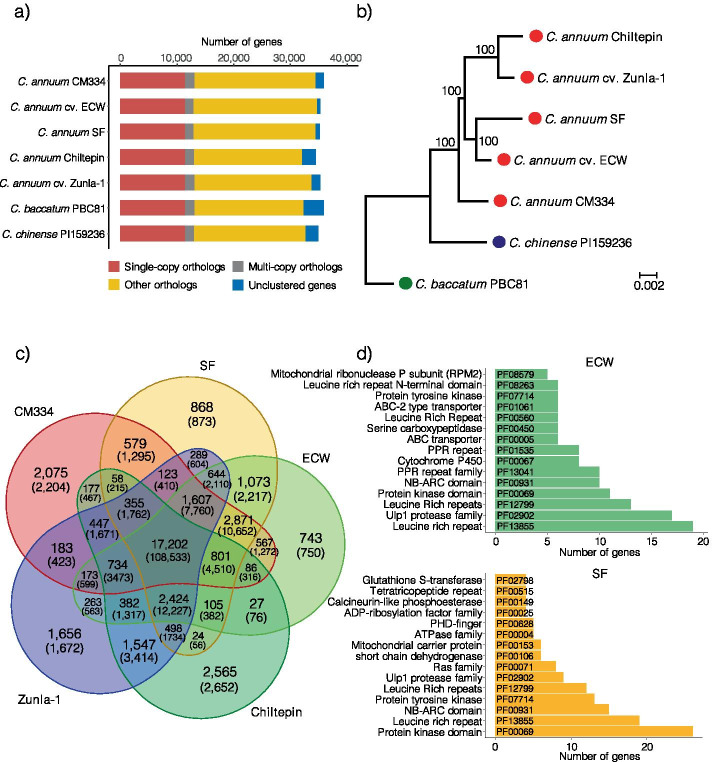
Gene family clustering and phylogenetic relationship among *Capsicum* species. **a** The number and types of orthologous genes. **b** The phylogenetic relationship between two novel (ECW and SF) and five publicly available pepper genome assemblies. **c** The number of shared and unshared clustered gene families between five *C. annuum* peppers. Total number of genes included in the clusters are shown in brackets. **d** The number of genes containing top 15 functional domains in unshared clusters of ECW and SF

Gene annotations from ECW, SF, and five publicly available *Capsicum* genomes (CM334, Zunla-1, and Chiltepin in *C. annuum*; PI159236 in *C. chinense*; and PBC81 in *C. baccatum*) were clustered into 35,037 groups. Subsequently, we classified the genes as single-copy (cluster containing one gene in all species), multi-copy (cluster containing more than one gene in all species), or other, based on the number of genes in each group (Fig. 1a and Supplementary Table 7). A total of 11,419 (32.6%) groups contained single-copy orthologs (Fig. 1a). ECW and SF had the smallest numbers of unclustered genes (541 [1.5%] and 627 [1.8%] genes, respectively), indicating that most of the protein sequences were very similar to those of other pepper gene annotations (Fig. 1a).

To further verify the evolutionary relationships, we constructed a phylogenetic tree using concatenation of the single-copy orthologs from pepper annotations (Fig. 1b). The four accessions in *C. annuum*, ECW, SF, Zunla-1, and Chiltepin, were more closely related to one another than to CM334 (Fig. 1b). A closer look at the gene clusters of *C. annuum* revealed that 108,533 genes (approximately 21,700 genes [61%] in each genome) were shared among 17,202 clusters (Fig. 1c). In the top 20 functional domain descriptions of genes in the core cluster, domains involved in defenses against pathogen and developmental functions were predominant (Supplementary Fig. 4).

On the other hand, ECW and SF contained 750 (2.1%) and 873 (2.5%) genes, respectively, which did not cluster with the other three accessions (Fig. 1c–d). Among them, genes containing NB-ARC, leucine-rich repeat, and protein kinase domains were most abundant (Fig. 1d and Supplementary Table 8). Gene ontology (GO) analyses of ECW and SF specific genes also demonstrated enrichment of disease resistance-related proteins such as late blight and TMV resistance proteins and LRR receptor-like kinases (Supplementary Fig. 5). Moreover, 2,204 (6.1%), 1,672 (4.7%), and 2,652 (7.7%) genes in CM334, Zunla-1, and Chiltepin, respectively, were not grouped with any accessions and also contained large numbers of defense-related domains against pathogens (Supplementary Fig. 6). Taken together, these observations indicate that specific gene repertoires, including disease resistance genes such as NLRs, have been dynamically changed among pepper genomes.

### Identification and classification of NLRs

**Fig. 2:**
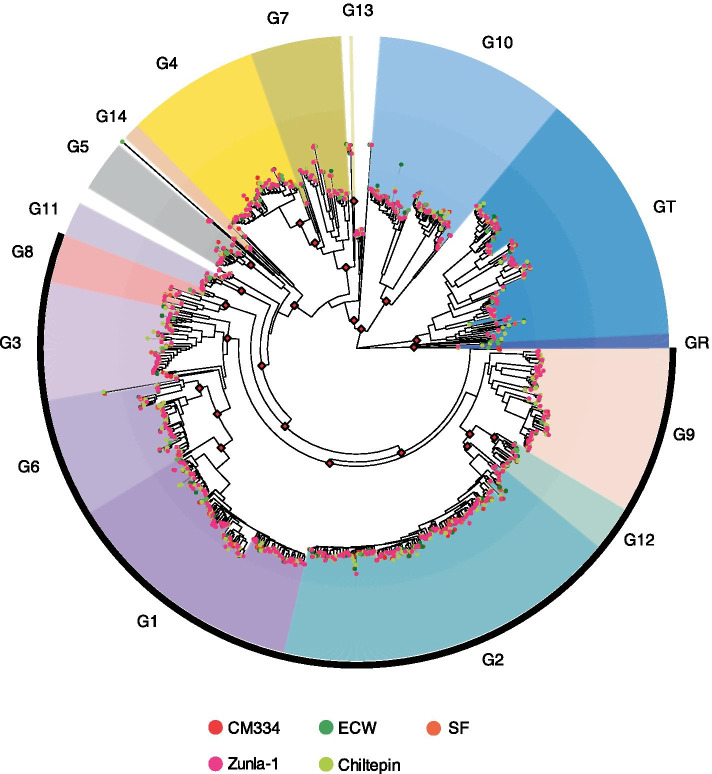
Phylogenetic relationship and classification of NLRs in *C. annuum.* The maximum-likelihood phylogenetic tree of NLRs from five pepper genomes was reconstructed using intact NB-ARC domains. Subgroups were assigned using ultra-fast bootstrap (UFBoot) and previous classification information [6, 21]. The branch between GR and GT subgroups was used as a root. NRC helper-dependent groups were marked as a black outline. Red diamonds at the nodes represent UFBoot values above 90

To elucidate NLR repertoires, we re-annotated NLRs in five pepper genomes (Fig. 2, Supplementary Table 9, and Supplementary Table 10). Between 760 (in ECW) and 972 NLRs (in Chiltepin) were identified (Supplemental Table 10). Among them, ECW and SF had the smallest numbers of NLRs, with 760 and 761, respectively, whereas CM334 and Chiltepin had the largest number of NLRs, with 951 and 972, respectively. Subsequently, we constructed a phylogenetic tree of NLRs and determined their subgroups. The analyses also revealed CNV of NLR subgroups among pepper accessions. In particular, G1 and G2, the largest subgroups in pepper NLRs [21], exhibited variable copy numbers among all accessions (Supplemental Table 10). On the other hand, GT, which includes TIR-NLR (TNL), and G10, referred to as the ancient and autonomous NLR (ANL) [22], had a moderate number of NLR subgroups and moderate CNVs. Moreover, GR, which includes RPW8-type helper NLR (RNL), and G8, which contains the NLRs required for cell death (NRC) helper group [23], had small numbers of NLRs and the fewest CNVs. Collectively, these results suggest that there are CNVs between accessions within the same NLR groups as well as between groups within a species.

### Intra-specific diversification of NLRs in pepper accessions

**Fig. 3:**
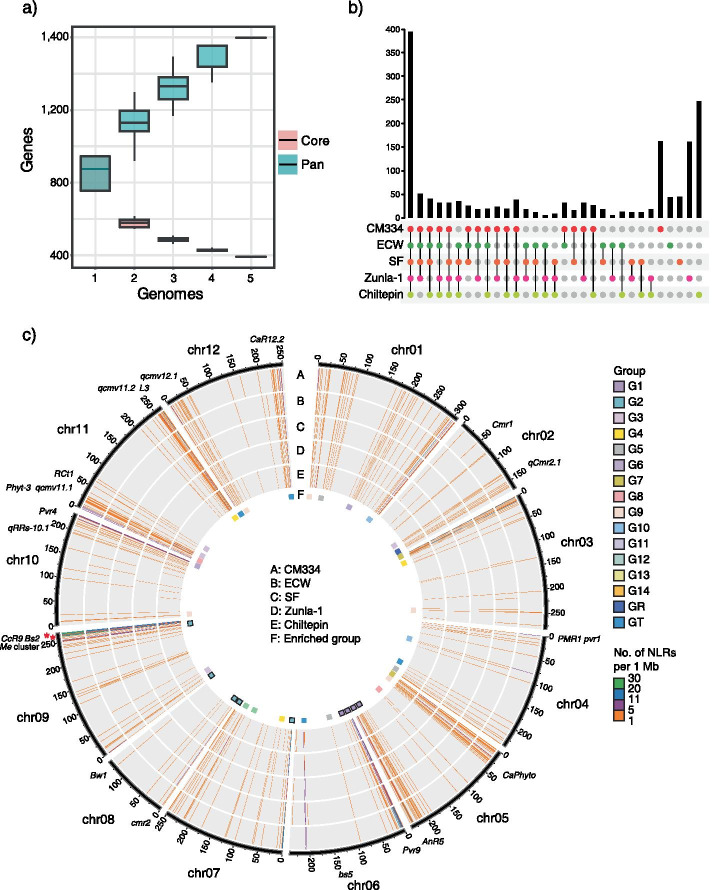
Construction of the NLR map in *C. annuum.* **a** The number of core- and pan- NLRs with increasing pepper accessions. **b** The distribution and orthologous relationship of core, dispensable, and specific NLRs among pepper genomes. **c** Chromosomal distribution of NLRs per 1 Mb window in CM334 genome as a reference. A-E: The heatmap represents NLR copy numbers of CM334, ECW, SF, Zunla-1, and Chiltepin, respectively, in each window. F: The enriched NLR subgroups in each window were marked as a rectangle with group colors. The subgroups G1 and G2 were marked with black borders. The two significant CNV regions located on chromosomes are marked with red asterisks

To detect accurate ortholog relationships of individual NLR genes among pepper accessions, we constructed an NLR map based on the CM334 reference genome and four other accessions (ECW, SF, Zunla-1, and Chiltepin) (Fig. 3 and Supplementary Table 11). As a result of this analysis, a total of 4,278 NLRs (98.2% of a total of 4,357) were assigned to the NLR map. The number of core NLR orthologs annotated in all accessions was 1,955 (391 pairs, 44.9%) (Fig. 3a). In addition to the core NLRs, a total of 1,670 dispensable NLRs were shared between two or more accessions (555 pairs, 38.3%), and the numbers of specific NLRs existing in only one accession were 161 (3.7%), 43 (1.0%), 44 (1.0%), 160 (3.7%), and 245 (5.6%) for CM334, ECW, SF, Zunla-1, and Chiltepin, respectively (Fig. 3b). The chromosomal distribution of NLRs based on the CM334 reference genome revealed that NLRs, including functional resistance genes, were enriched at both ends of chromosomes, and that subgroups were located on specific chromosomes (Fig. 3c). For example, NLRs in G1 and G2 were enriched at chromosomes 5 and 9, respectively (Supplementary Table 12).

**Fig. 4:**
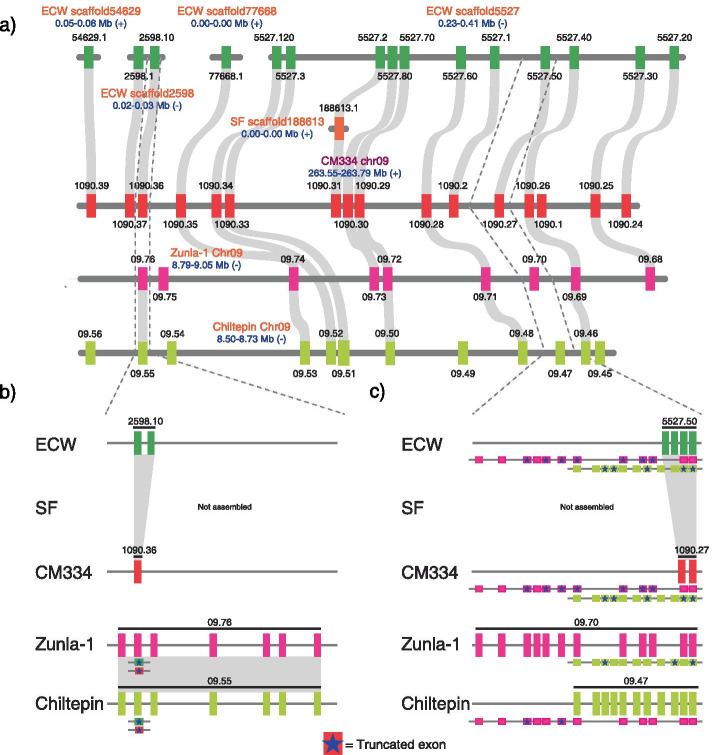
Detailed comparison of the syntenic CNV region. **a** The NLRs of chr09:263.55–263.79 Mb in CM334 and corresponding regions in other accessions were depicted. Each rectangle and grey curves represent the position of NLR genes and orthologous relationships, respectively. **b** and **c** Diversification of CNVs in NLRs. Each rectangle and black line represent an exon and gene boundary, respectively. The exons containing early stop codons were marked with a blue star

To identify CNV regions in which NLRs were not evenly distributed among pepper accessions, we performed a chi-square test on the number of NLRs in physical clusters where intergenic region of NLRs was less than 50 kb. Two chromosomal regions and three scaffolds exhibited significant CNVs in NLR (Supplementary Table 13, adjusted *P*-value < 0.05). For example, when we compared 16 NLRs of the chr09:263.55–263.79 Mb genomic region enriched in CM334 in the G2 subgroup with the corresponding regions of all other accessions, all 15 NLRs in ECW and seven NLRs out of nine NLRs in Zunla-1 had orthologous genes in at least one other accession. By contrast, we identified only one NLR in the SF genome and seven out of 12 NLRs in Chiltepin with orthologous genes in the same region (Fig. 4a). These results indicate that extreme CNVs of NLRs could be the result of low NLR copy numbers in the SF and Chiltepin genomes.

Specifically, CA.PGAv.1.6.scaffold1090.36 in CM334, ECW.scaffold2598.10 in ECW, Chr09.76 in Zunla-1, and Chr09.55 in Chiltepin did not match the NLRs in SF because the corresponding region was omitted during SF genome assembly. The NLRs of CA.PGAv.1.6.scaffold1090.36 and ECW.scaffold2598.10 also did not match the NLRs of Chr09.76 in Zunla-1 and Chr09.55 in Chiltepin because large insertions of 7,841 bp and 8,113 bp were located in Zunla-1 and Chiltepin genomes, respectively. Consequently, orthologous relationship between those genes were broken (Fig. 4b). When we mapped CA.PGAv.1.6.scaffold1090.36 in CM334 and ECW.scaffold2598.10 in ECW to the Zunla-1 and Chiltepin genomic regions, we identified early stop codons due to point mutation that generated abnormal termination of translation (Fig. 4b). In another case, we identified 14 stop codons in CM334 and ECW genomic regions corresponding to Chr09.70 in Zunla-1 (Fig. 4c). These stop codons were the consequence of both point mutations and frameshifts resulting from a combination of insertions or deletions (InDels). Mapping the Chr09.47 protein of Chiltepin to similar genomic regions in CM334 and ECW revealed six stop codons resulting from point mutations and insertions. Instead, an NLR in each of the genomes were annotated between the regions where the Chr09.70 and Chr09.47 proteins were mapped. When Chr09.70 and Chr09.47 were mapped to each genome, we identified three and six stop codons, respectively, resulting from point mutations. PCR amplification and Sanger sequencing also confirmed that small variation-mediated early stop codons resulted in truncated proteins of NLRs (Supplementary Fig. 7). Therefore, small genomic variations such as point mutation and InDels caused changes in the NLR repertoire. Taken together, these results suggest that small sequence variations mediate the CNVs of NLRs within a species.

## Discussion

Recently, plant pan-genomes have replaced the role of reference genomes [13]. However, the limited number of high-quality de novo genome assemblies, especially for large plant genomes, hinders the implementation of their pan-genome studies. Specifically, the pan-genome of pepper constructed based on low-depth sequencing approaches [24] is still limited in understanding genomic diversity among pepper genomes. Here, we presented the genome assemblies with annotations of two pepper accessions, ECW and SF, containing large contig N50 of over 100 kb via short-read sequencing (Table 1). The assessments of genome assemblies and annotations using BUSCO revealed that quality of two de novo genome assemblies and annotations are adequate for comparing to the other publicly available genomes for further analyses (Supplementary Table 4). The phylogenetic tree from single-copy orthologous genes was slightly different from a previous study [4] probably because of the application of different methodology such as maximum-likelihood and neighbor-joining methods resulted in different topology (Fig. 1b). Nevertheless, the closest relationship between Zunla-1 and its wild progenitor Chiltepin suggested that our phylogenetic tree represents reasonable topology. These results indicate that these two newly assembled and annotated genomes are of sufficient enough quality to compare gene repertoires with other genome assemblies and construct chromosome level assembly for pan-genome in *C. annuum*.

In general, annotation bias could be generated by different annotation methods and resources and prevent accurate comparative analyses of genes. In this study, re-annotation of NLRs was performed using the same method [25] with same protein sequences and finally could provide improved NLR resources in the five pepper genomes. Phylogenetic and comparative genomic analyses suggested that the G1 and G2 subgroups, which had been greatly duplicated in pepper [21], were diversified with large CNVs, not only after speciation but also after divergence within species (Supplementary Table 9 and 10). Conversely, the GR and G8 subgroups were conserved in all accessions (Supplementary Table 9 and 10). Because these groups contain helper NLRs that interact with multiple sensor NLRs to recognize pathogen effectors and mediate immune signaling [23], the evolution of NLRs in these groups may be more stringently regulated and conserved. Furthermore, we constructed an NLR map using whole-genome alignment to accurately predict the orthologous relationship of NLRs in *C. annuum* (Fig. 3). Comparative analysis based on the NLR map identified regions in which CNVs of NLRs differed significantly in *C. annuum*. This phenomenon has also been observed in other species, including *Arabidopsis* and tomato [18, 19]. However, the detailed comparison of the CNV region revealed that truncated protein structures of NLRs mediated CNVs due to genomic sequence variations such as InDels and other mutations (Fig. 4). These results indicate that small genomic variations are crucial to the evolutionary process for NLR diversification.

We identified five statistically significant CNV regions which apparently appears to be a small number compared to more significant CNVs detected in the NLR groups based on the phylogenetic tree (Supplementary Table 10 and 13). This was due to the limited number of pepper genomes used to construct the NLR map. Nevertheless, analysis of NLR gene family integrated with phylogeny, synteny, and statistical test could provide comprehensive understanding of NLR diversity. Recently, a pan-genome analysis using 14 multiple reference genomes and 100 diverse lines in tomato elucidated the relationship between the number of copies and functional variations of genes such as *NON-SMOKY GLYCOSYL TRANSFERASE1* (*NSGT1*) and *NSGT2* [16]. This suggests that comprehensive analyses of NLRs combined with multiple strategies and more genome assemblies could detect more CNVs and elucidate the evolutionary and functional mechanisms of NLRs associated with genomic variation.

## Conclusion

In conclusion, we assembled and annotated two pepper genomes and construction of NLR map in pepper with publicly available genomes revealed the CNVs of NLR gene family. These two new pepper genome assemblies, annotations, and the NLR map represent a valuable resource for identification of functional disease resistance genes, as well as for studying the evolutionary mechanisms of disease resistance in genus *Capsicum*.

## Materials and methods

### DNA extraction and sequencing

Since the pepper reference genome (CM334) is a landrace close to a wild species, two pepper accessions were selected to generate basic resources for pan-genome analysis. The accessions used in this study were ‘Early Calwonder (ECW, IT158295)’, a non-pungent, bell-shaped pepper, and ‘Small Fruit (SF, IT218615)’, a pepper with a high content of capsaicinoids. In addition, the cultivar ECW is known to be susceptible to the bacterial spot pathogens (*Xanthomonas* spp.) and used as near-isogenic lines for bacterial spot resistance genes (*Bs1, Bs2, Bs3, Bs4,* and *bs6*) [26, 27]. Both were obtained from the RDA-Genebank Information Center of National Agrobiodiversity Center (NAAS, RDA, Republic of Korea). The plants were grown at 24 °C under a 16/8 h light/dark cycle in an environmentally controlled growth chamber. Leaves from 3-week-old plants were frozen immediately in liquid nitrogen for isolation of genomic DNA. Genomic DNA (gDNA) with high molecular weight was extracted from frozen leaves, and the quality of gDNA was confirmed by spectrophotometric analysis (DS-11 Spectrophotometer; DeNovix Inc.) and agarose gel electrophoresis (1.0% *w/v* agarose TAE 1X gel containing 1X EcoDye; BIOFACT, Daejeon, Korea). The paired-end (PE) and mate-pair (MP) libraries for NGS were constructed using the TruSeq DNA Nano Kit for 350-, 550-, and 600–800 bp insert sizes and the Nextera Mate Pair Kit for 2- and 5-kb insert sizes, respectively (Illumina, San Diego, CA, USA). The quality of each library was validated by qPCR. PE and MP libraries were sequenced with the HiSeqX-ten and NovaSeq6000 sequencing platforms, respectively (Illumina).

### De novo genome assembly

A total of 460.2 Gb (146.8 ×) of ECW and 460.2 Gb (145.8 ×) of SF raw data were pre-processed using the “quality_trim” (-q 20 –m 76) and “remove_duplicated” functions implemented in CLC tools v4.0.6 (CLC bio, Aarhus, Denmark) to remove low-quality and duplicated sequences. To estimate genome size, the 19-mer frequency was calculated using Jellyfish v1.1.5 [28] to estimate genome size. Among the filtered PE libraries, short reads with overlaps were merged into longer fragments by FLASH v1.2.2 (–m 30 –M 100 –x 0.1 –r 151 –f 300 –s 40) [29], and then assembled into initial contigs with Platanus v1.2.4 (–k 71 –c 5 –d 0.3 –t 60 –m 750) [30]. With the addition of MP libraries, scaffold assembly was also performed by Platanus (–l 3 –s 51 –u 0.2 –t 30). Assembly gaps were closed with Platanus (–ed 0.1 –t 30) using reads from both libraries.

### Gene and repeat annotation

Gene annotation of the two pepper genomes was performed as described in Kim et al. [6], except for transcript annotation for the SF genome. For annotation of the ECW genome, RNA-seq reads from fruit tissues [3] were aligned to the assembled genome using TopHat v2.1.1 [31] and Cufflink v2.2.1 [32] with default settings to build transcripts, which were processed with ISGAP [33] to identify coding sequences. Publicly available protein sequences of *C. annuum* CM334 v2.0 [5], cv. Zunla-1 v2.0 [4], var. *glabriusculum* Chiltepin v2.0 [4], *C. baccatum* PBC81 v1.2 [6], *C. chinense* PI159236 v1.2 [6], and *Solanum lycopersicum* ITAG3.2 [34] were mapped to the ECW and SF genomes using Exonerate v2.2.0 [35]. *Ab-initio* prediction was performed with Augustus v3.2.3 [36] using a previously constructed training set for the pepper genome [6]. Subsequently, the transcriptome, protein alignment and *ab-initio* prediction were merged to complete the final gene model for ECW; only the latter two were merged for SF. The functional annotations of these gene models were generated with InterproScan v5.22–61.0 (-f tsv -iprlookup –goterms –appl TIGRFAM, ProDom, SMART, ProSiteProfiles, ProSitePatterns, SUPERFAMILY, PRINTS, Pfam) [37] and BLASTp (-evalue 1e-4 –max_target_seqs 5) using publicly available annotation databases, including RefSeq [38] and UniProt/Swiss-Prot [39]. To validate genome assemblies and gene annotations, we performed BUSCO v3.1.0 [20] with 1,375 conserved ortholog proteins in Embryophyta (odb10). We also compared the length distributions of genes, exons, introns, and CDSs between published pepper gene annotations [4, 6].

After gene annotation was completed, repeat annotation was performed for both pepper genomes by RepeatMasker v4.0.3 (http://www.repeatmasker.org) with default options and the pepper genome repeat library constructed in the previous study [6].

### Identification of orthologous group and phylogenetic analysis

Protein sequences were clustered from seven peppers, including two new annotations from this study and five published annotations of *C. annuum* CM334 [6], cv. Zunla-1 [4], Chiltepin [4], *C. baccatum* PBC81 [6], and *C. chinense* PI159236 [6]. Single-copy orthologs were concatenated and aligned using OrthoFinder v2.2.7 (-M msa) [40]. The alignment of single-copy orthologs was imported to construct a maximum likelihood tree using IQ-TREE v1.6.12 (-alrt 1000 -bb 1000 -nt AUTO -safe -blmin 10e-6) [41]. The best substitution model was selected as VT + F + R2 with ModelFinder [42] implemented in IQ-TREE. Ortholog copies in the *C. annuum* species were compared and visualized as a Venn diagram using TBtools v1.051 [43]. Of these, Pfam domain contents were extracted except for transposable element-related domains from unclustered genes in *C. annuum* for functional comparison. Gene ontology (GO) term enrichment analyses for those unclustered genes were performed by comparing to total genes in each accession using Blast2GO [44].

### Identification and classification of NLR genes

To identify additional NLRs, we re-annotated each pepper genome assembly using TGFam-Finder v1.20 with default parameter [25]. Briefly, we used the same genome assemblies and annotations described above for *C. annuum* (CM334, ECW, SF, Zunla-1, and Chiltepin) and searched domains. After six-frame translation of the genomes, the target regions containing NB-ARC domain (PF00931) with 100 kb flanking sequence were searched. The NLRs of 50 plants used by Kim et al. [25], and each pepper annotation containing NB-ARC domains were used as resource proteins for protein mapping. RNA-seq reads obtained by the previous report [3] were used for transcriptome mapping. *Ab-initio* gene prediction was performed and final gene models were generated by combining gene models from protein alignments, assembled transcripts, and *ab-initio* gene prediction.

To assign putative NLR groups, the pipeline of NLR classification established by previous studies [6, 19, 21] was used with some modifications. Known NLR genes from GenBank and Plant Resistance Genes database (PRGdb) v3.0 (Supplementary Table 1) [45], and NLR group information from Kim et al. [6], were used as references for group assignment. The NB-ARC domains of NLRs were searched and extracted using NLR-parser v1.0 (*P*-value cutoff = 1.9e-5) [46]. We defined an intact NB-ARC domain with at least three of four major motifs (P-loop, GLPL, Kinase2, and MHDV) placed in sequence order and a length of at least 160 amino acids. These intact NB-ARC domains were aligned using MAFFT v7.407 (–maxiterate 1000 –globalpair) [47] and positions with gaps above 92% in aligned sequences were removed using trimAl v1.4.rev22 [48]. A maximum-likelihood phylogenetic relationships were inferred from IQ-TREE v1.6.12 [41] with ultra-fast bootstrap (UFBoot) [49] of 1000 (-bb 1000 –alrt 1000 -safe). The substitution model was selected with ModelFinder [42] implemented in IQ-TREE. The best-fit model was JTT + F + R7. The group of intact NLRs was assigned based on known NLR genes, UFBoot value > 90% and previously assigned group information [6]. For partial NLRs without an intact NB-ARC domain, a putative NLR group was assigned using the group of intact NLRs and BLASTp (-evalue 1e-4). The group with the highest number of matches above 50% similarity and 30% coverage versus the NB-ARC domain of intact NLRs was assigned to partial NLRs.

### Construction of NLR map and extraction of regions for CNV analysis

The NLR map was constructed using ppsPCP v1.0 with default parameter [50]. The putative positions of NLRs were assigned using output from NUCmer and delta-filter (-1 option) implemented in MUMmer v4.0.0beta2, which is the part of the ppsPCP pipeline. Genes that were neither anchored to the NLR map nor specific to each accession were filtered for downstream analysis. NLRs that overlapped within 50 kb were defined as physical cluster using the “merge” function implemented in bedtools v2.25.0 [51]. Based on physical clustering, orthologous relationships were predicted using iteration of get_homologues-est v1.0 with -M -c -A -t 0 options [52]. Box and upset plots were visualized using ggplot [53] and TBtools v1.051 [43], respectively. The physical NLR clusters (standard deviation > 2) containing significant CNVs were detected by chi-square test with false discovery rate (FDR) correction in the “chisq_test” function in rstatix (https://cran.r-project.org/web/packages/rstatix/index.html) v0.6.0 and “p.adjust” function in R (https://www.R-project.org/) v3.6.3, respectively. Enrichment and FDR correction of NLR subgroups were performed using Perl modules "Math::GSL::CDF” (https://metacpan.org/pod/Math::GSL::CDF) and "Statistics::Multtest" (https://metacpan.org/pod/Statistics::Multtest), respectively. The NLR map was plotted using Circos v0.69–9 [54]. Based on significant CNV regions in the NLR map, the NLRs of syntenic regions in chromosomes or scaffolds for each pepper accession were extracted and plotted using “jcvi.graphics.synteny” function implemented in the JCVI package [55] and simplified using Illustrator.

### Confirmation of CNVs in NLRs via PCR amplification and Sanger sequencing

PCR amplification was performed using gDNA from CM334, ECW, and Zunla-1. Two primer sets of CDS regions of 1) CA.PGAv.1.6.scaffold1090.36, ECW.scaffold2598.10, and syntenic segment in Zunla-1 and 2) Chr09.70 in Zunla-1 and corresponding segments in CM334 and ECW were designed as follows: 5’-CAGTTCCCACAAGAAGCTAAAAGAC-3’, 5’-GTTAAATGAGCTAAAGCTACTGAGTTTTTTG-3’ for amplifying CA.PGAv.1.6.scaffold1090.36 in Zunla-1 and 5’-CAGCAACGTAGAAAACAATACCTAAG-3’, 5’-CACCATATAAATGCACGACAATAGTTAG-3’ in CM334 and ECW; 5’-CCTTGATTGATGCCGAGATTAG-3’, 5’-GAATAGAGTGTTCAATGATATTCTGATC-3’ for amplifying Chr09.70 in Zunla-1 and 5’-CCACTTACTAAATTGACTCAGAAAAAG-3’, 5’-CCTTACTCTATACTCAAATTTTCTACC-3’ in CM334 and ECW. Specificity of each primer set was confirmed by BLASTn search (-evalue 1). PCR condition (3 min 98 °C heat start; 30 s, 98 °C denaturation step; 15 s, 58 °C annealing step; 70 s, 68 °C extension step with 35 cycles) was modified from basic fast 3-step PCR protocol of commercial Primestar GXL (TAKARA®) enzyme mixture. PCR products were loaded in 1% agarose gel and purified using commercial kit (Cosmo GENETECH®) for Sanger sequencing.

## Supplementary Information

**Additional file 1: Figure S1.** Outline of the genome assembly and annotation workflow. **Figure S2.** Distribution of 19-mer frequency in two pepper cultivars. **Figure S3.** Comparison of gene models of five different pepper cultivars. **Figure S4.** The top 20 highest number of genes containing functional domains shared by CM334, ECW, SF, Zunla-1, and Chiltepin in *Capsicum annuum*. **Figure S5.** Gene ontology enrichment analyses of unclustered genes in pepper accessions. **Figure S6.** The number of genes containing functional domains specific to CM334, Chiltepin, and Zunla-1. **Figure S7.** PCR and sequencing validation for CNV of NLRs.

**Additional file 2: Table S1.** Reference disease resistance genes used in this study. **Table S2.** Statistics of raw data used in this study. **Table S3.** K-mer frequency of ECW and SF. **Table S4.** Validation of genome assembly and gene annotation using BUSCO. **Table S5.** Comparison of predicted genes using published annotation database. **Table S6.** Summary of repeat annotation. **Table S7.** Number of orthologs per species. **Table S8.** Number of specific genes containing functional domains of ECW and SF in *C. annuum.***Table S9.** Assigned NLR group and NB-ARC type. **Table S10.** Statistics of classified NLRs in *C. annuum.***Table S11.** Ortholog pair of NLRs between five accessions in *C. annuum.***Table S12.** Statistics of enriched group of NLRs per 1 Mb window. **Table S13.** List of significant CNV regions for NLRs.

## Data Availability

The sequenced raw data were deposited into the NCBI Sequence Read Archive (SRA). Accession numbers are SRP119199 for both ECW (SRR10007904 to SRR10007908) and SF (SRR10007830 to SRR10007834). The final assembled genomes and annotations are available at GenBank under the accession numbers VYZY01000000 (ECW) and VYZZ01000000 (SF), and can be downloaded from our website (http://peppergenome.snu.ac.kr). The additional data set and scripts were deposited in GitHub (https://github.com/sdaf11111/NLR-map-in-pepper).

## Abbreviations

ANL: Ancient and autonomous NLR
CM334: Criollo de Morelos-334
CNV: Copy number variation
ECW: Early Calwonder
InDel: Insertion or deletion
MP: Mate-pair
MYA: Million years ago
NB-ARC: Nucleotide-binding
NGS: Next-generation sequencing
NLR: Nucleotide-binding and leucine-rich repeat gene
NRC: NLR required for cell death
PE: Paired-end
RNL: RPW8-type helper NLR
SF: Small Fruit
SNP: Single-nucleotide polymorphism
TNL: Toll/IL-1 receptor-NLR
