# Supplementary material for: Comparative analysis of de novo genomes reveals dynamic intra-species divergence of NLRs in pepper
Source: BMC Plant Biol. 2021 May 31;21:247. doi: 10.1186/s12870-021-03057-8 (PMC8166135; doi:10.1186/s12870-021-03057-8)
Supplement: Supplementary file 1 — Additional file 1: Figure S1. Outline of the genome assembly and annotation workflow. Figure S2. Distribution of 19-mer frequency in two pepper cultivars. Figure S3. Comparison of gene models of five different pepper cultivars. Figure S4. The top 20 highest number of genes containing functional domains shared by CM334, ECW, SF, Zunla-1, and Chiltepin in Capsicum annuum. Figure S5. Gene ontology enrichment analyses of unclustered genes in pepper accessions. Figure S6. The number of genes containing functional domains specific to CM334, Chiltepin, and Zunla-1. Figure S7. PCR and sequencing validation for CNV of NLRs. [file 12870_2021_3057_MOESM1_ESM.pdf]

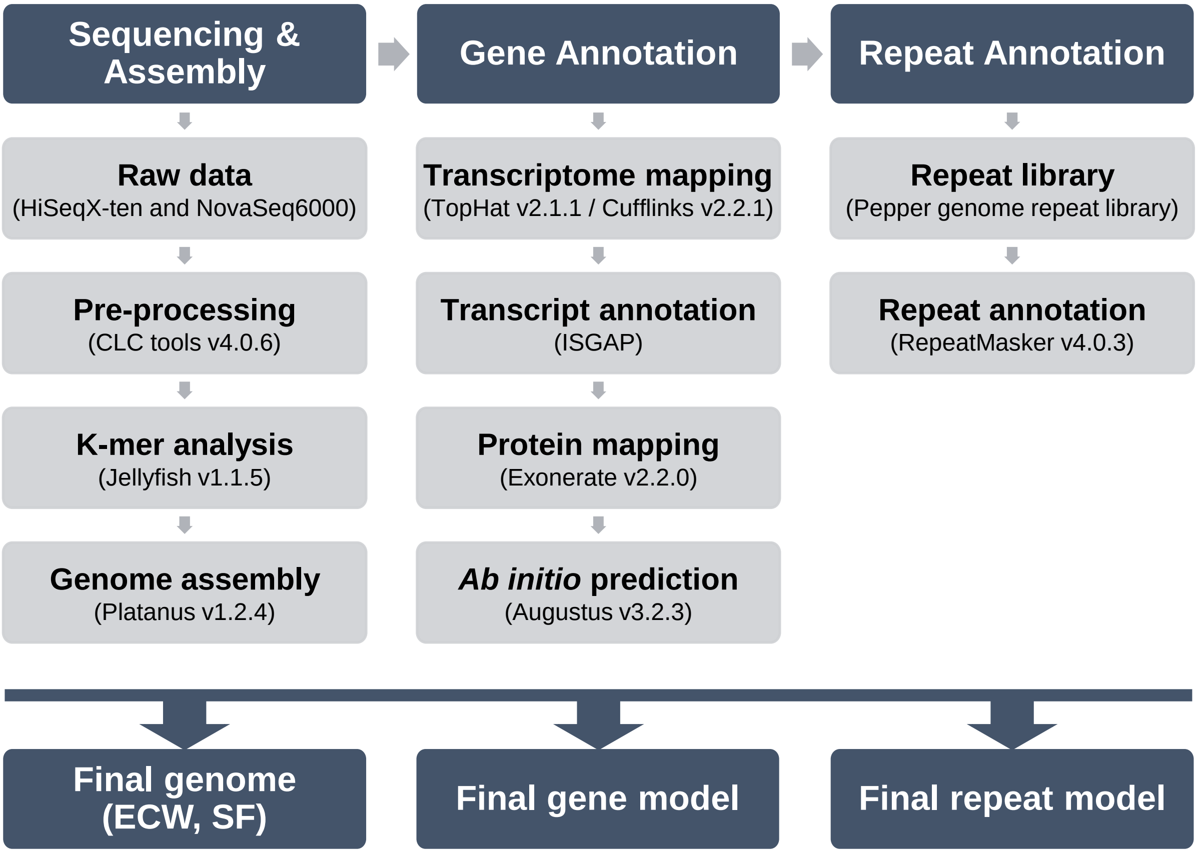

Supplementary Figure 1. Outline of the genome assembly and annotation workflow. Low quality and duplicated sequences were trimmed and removed from the raw data. The distribution of 19-mer frequency and *de novo* genome assembly were performed. Structural gene annotations were conducted via transcriptome-based annotation, protein mapping using publicly available resources, and *ab initio* predictions. The final gene models were generated after integration of genes from each annotation process. Subsequently, repeats were annotated using previously constructed repeat library of pepper genome (Kim *et al.* 2017). See Methods section for detailed information on tools and parameters.

a)

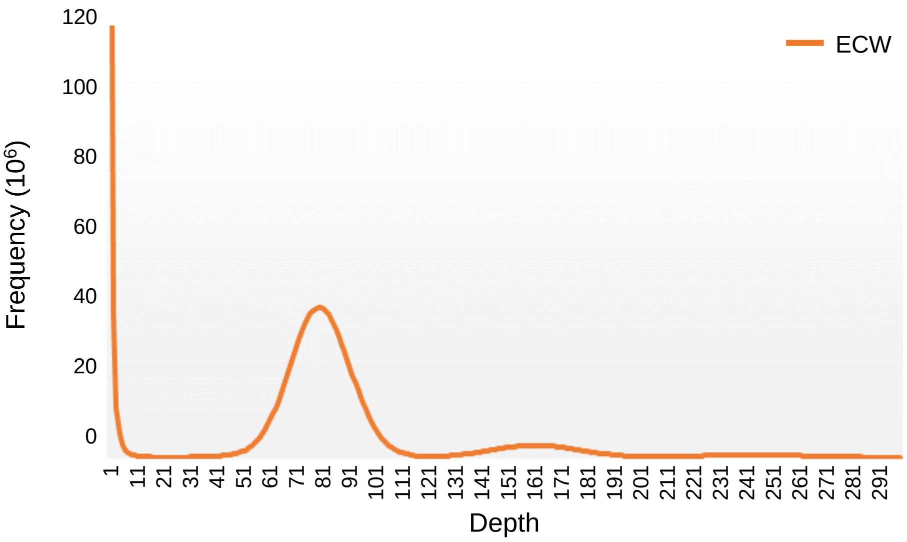

b)

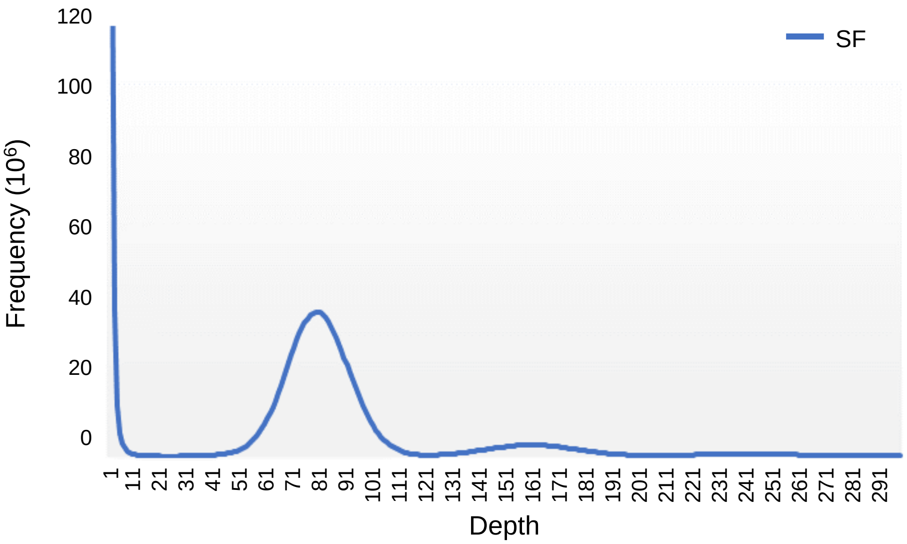

Supplementary Figure 2. Distribution of 19-mer frequency in two pepper cultivars. The 19-mer frequency of filtered sequences for a) ‘Early Calwonder (ECW)’ and b) ‘Small Fruit (SF)’ was calculated for estimation of genome size. The main peaks are located at 80. The x-axis is labelled as the depth of 19-mers and the y-axis shows the volume of each 19-mer frequency.

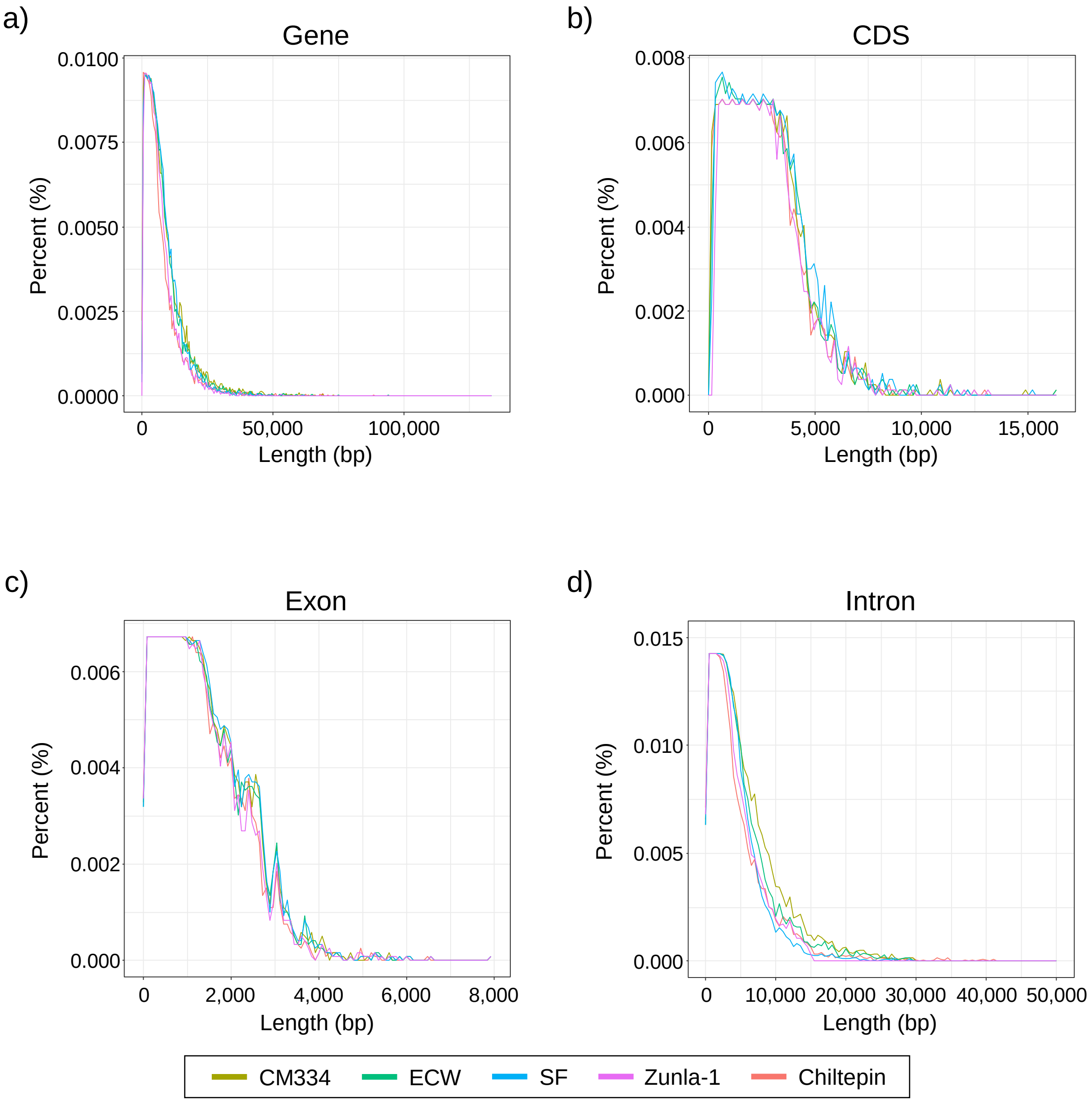

Supplementary Figure 3. Comparison of gene models of five different pepper cultivars. The length distribution of a) gene, b) CDS, c) exon and d) intron were compared between *Capsicum annuum* CM334, ECW, SF, Zunla-1, and Chiltepin.

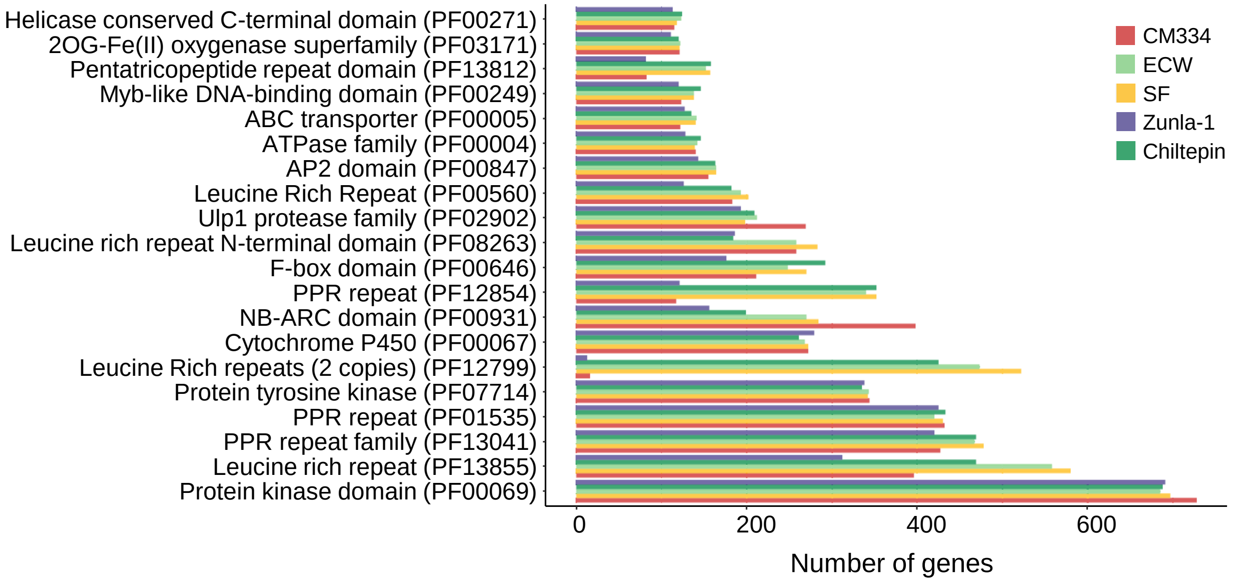

Supplementary Figure 4. The top 20 highest number of genes containing functional domains shared by CM334, ECW, SF, Zunla-1, and Chiltepin in *Capsicum annuum*.

a)

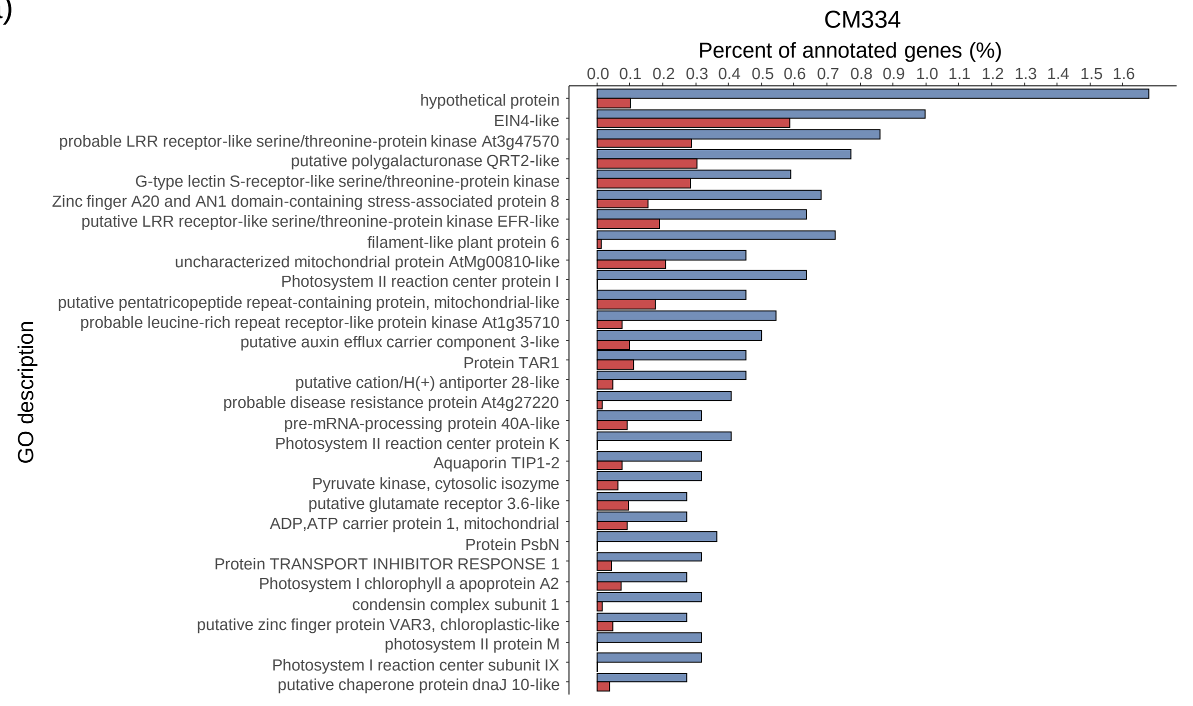
CM334
Percent of annotated genes (%)
0.0 0.1 0.2 0.3 0.4 0.5 0.6 0.7 0.8 0.9 1.0 1.1 1.2 1.3 1.4 1.5 1.6
GO description
hypothetical protein
EIN4-like
probable LRR receptor-like serine/threonine-protein kinase At3g47570
putative polygalacturonase QRT2-like
G-type lectin S-receptor-like serine/threonine-protein kinase
Zinc finger A20 and AN1 domain-containing stress-associated protein 8
putative LRR receptor-like serine/threonine-protein kinase EFR-like
filament-like plant protein 6
uncharacterized mitochondrial protein AtMg00810-like
Photosystem II reaction center protein I
putative pentatricopeptide repeat-containing protein, mitochondrial-like
probable leucine-rich repeat receptor-like protein kinase At1g35710
putative auxin efflux carrier component 3-like
Protein TAR1
putative cation/H(+) antiporter 28-like
probable disease resistance protein At4g27220
pre-mRNA-processing protein 40A-like
Photosystem II reaction center protein K
Aquaporin TIP1-2
Pyruvate kinase, cytosolic isozyme
putative glutamate receptor 3.6-like
ADP,ATP carrier protein 1, mitochondrial
Protein PsbN
Protein TRANSPORT INHIBITOR RESPONSE 1
Photosystem I chlorophyll a apoprotein A2
condensin complex subunit 1
putative zinc finger protein VAR3, chloroplastic-like
photosystem II protein M
Photosystem I reaction center subunit IX
putative chaperone protein dnaJ 10-like

b)

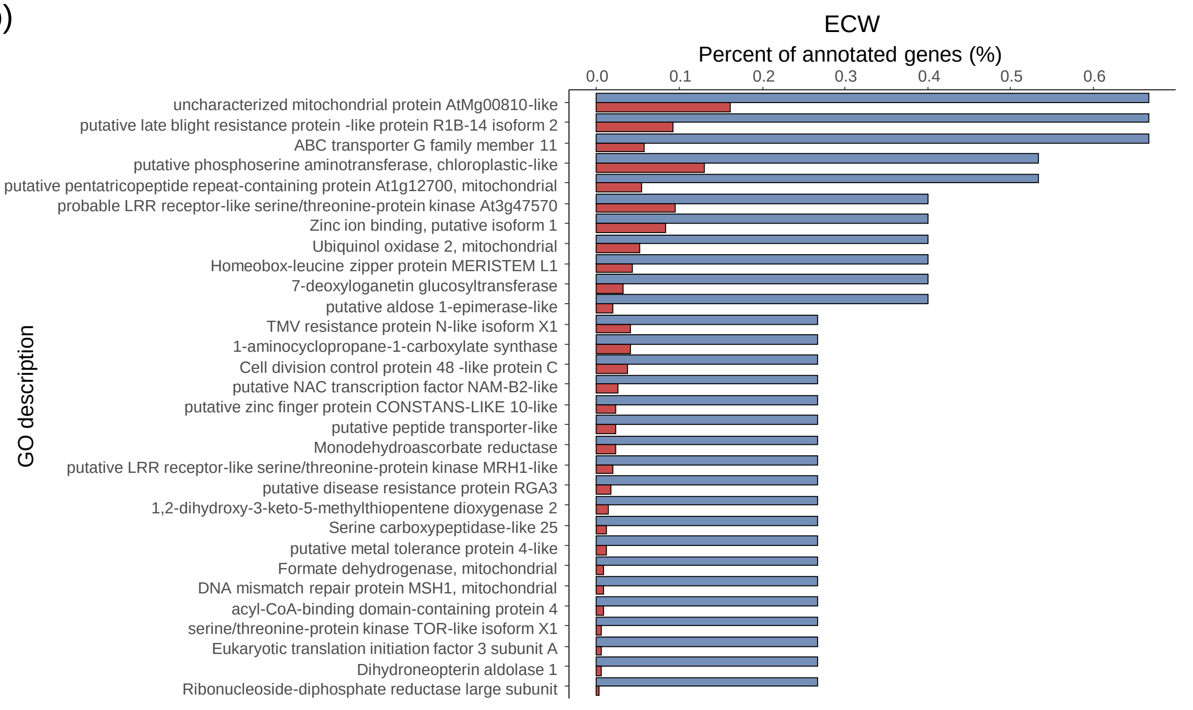
ECW
Percent of annotated genes (%)
0.0 0.1 0.2 0.3 0.4 0.5 0.6
GO description
uncharacterized mitochondrial protein AtMg00810-like
putative late blight resistance protein -like protein R1B-14 isoform 2
ABC transporter G family member 11
putative phosphoserine aminotransferase, chloroplastic-like
putative pentatricopeptide repeat-containing protein At1g12700, mitochondrial
probable LRR receptor-like serine/threonine-protein kinase At3g47570
Zinc ion binding, putative isoform 1
Ubiquinol oxidase 2, mitochondrial
Homeobox-leucine zipper protein MERISTEM L1
7-deoxyloganetin glucosyltransferase
putative aldose 1-epimerase-like
TMV resistance protein N-like isoform X1
1-aminocyclopropane-1-carboxylate synthase
Cell division control protein 48 -like protein C
putative NAC transcription factor NAM-B2-like
putative zinc finger protein CONSTANS-LIKE 10-like
putative peptide transporter-like
Monodehydroascorbate reductase
putative LRR receptor-like serine/threonine-protein kinase MRH1-like
putative disease resistance protein RGA3
1,2-dihydroxy-3-keto-5-methylthiopentene dioxygenase 2
Serine carboxypeptidase-like 25
putative metal tolerance protein 4-like
Formate dehydrogenase, mitochondrial
DNA mismatch repair protein MSH1, mitochondrial
acyl-CoA-binding domain-containing protein 4
serine/threonine-protein kinase TOR-like isoform X1
Eukaryotic translation initiation factor 3 subunit A
Dihydroneopterin aldolase 1
Ribonucleoside-diphosphate reductase large subunit

c)

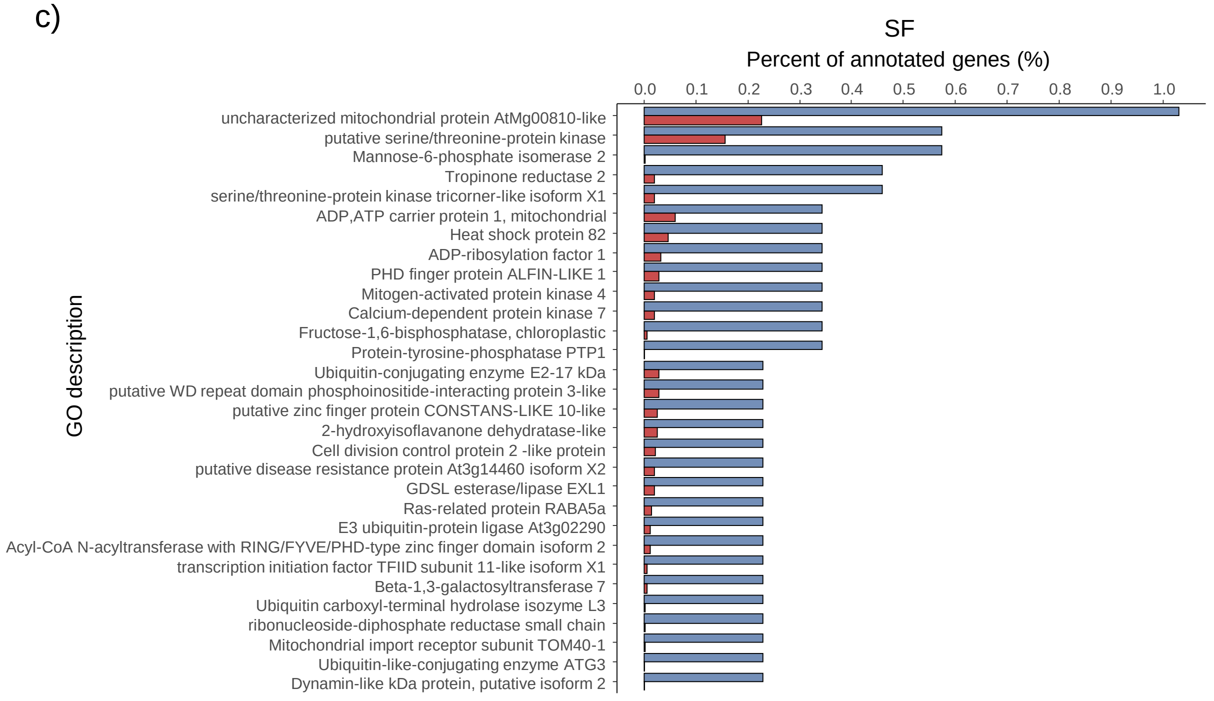
SF
Percent of annotated genes (%)
0.0 0.1 0.2 0.3 0.4 0.5 0.6 0.7 0.8 0.9 1.0
GO description
uncharacterized mitochondrial protein AtMg00810-like
putative serine/threonine-protein kinase
Mannose-6-phosphate isomerase 2
Tropinone reductase 2
serine/threonine-protein kinase tricorner-like isoform X1
ADP,ATP carrier protein 1, mitochondrial
Heat shock protein 82
ADP-ribosylation factor 1
PHD finger protein ALFIN-LIKE 1
Mitogen-activated protein kinase 4
Calcium-dependent protein kinase 7
Fructose-1,6-bisphosphatase, chloroplastic
Protein-tyrosine-phosphatase PTP1
Ubiquitin-conjugating enzyme E2-17 kDa
putative WD repeat domain phosphoinositide-interacting protein 3-like
putative zinc finger protein CONSTANS-LIKE 10-like
2-hydroxyisoflavanone dehydratase-like
Cell division control protein 2 -like protein
putative disease resistance protein At3g14460 isoform X2
GDSL esterase/lipase EXL1
Ras-related protein RABA5a
E3 ubiquitin-protein ligase At3g02290
Acyl-CoA N-acyltransferase with RING/FYVE/PHD-type zinc finger domain isoform 2
transcription initiation factor TFIID subunit 11-like isoform X1
Beta-1,3-galactosyltransferase 7
Ubiquitin carboxyl-terminal hydrolase isozyme L3
ribonucleoside-diphosphate reductase small chain
Mitochondrial import receptor subunit TOM40-1
Ubiquitin-like-conjugating enzyme ATG3
Dynamin-like kDa protein, putative isoform 2

d)

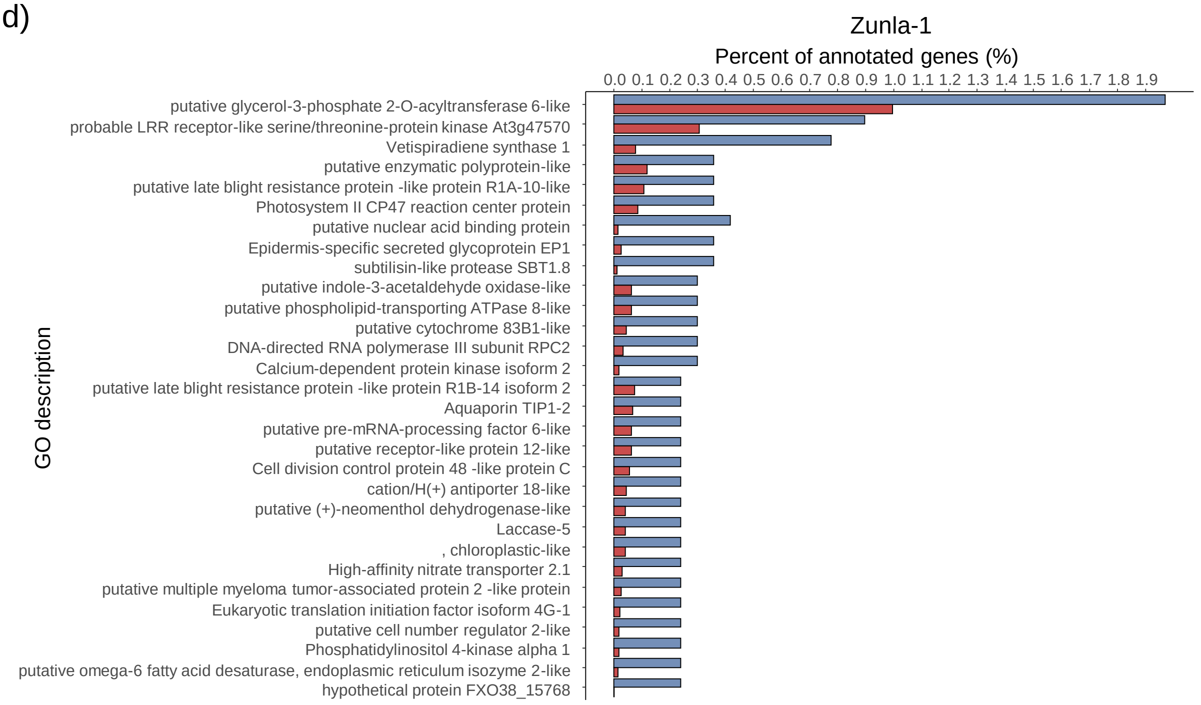
Zunla-1
Percent of annotated genes (%)
0.0 0.1 0.2 0.3 0.4 0.5 0.6 0.7 0.8 0.9 1.0 1.1 1.2 1.3 1.4 1.5 1.6 1.7 1.8 1.9
GO description
putative glycerol-3-phosphate 2-O-acyltransferase 6-like
probable LRR receptor-like serine/threonine-protein kinase At3g47570
Vetispiradiene synthase 1
putative enzymatic polyprotein-like
putative late blight resistance protein -like protein R1A-10-like
Photosystem II CP47 reaction center protein
putative nuclear acid binding protein
Epidermis-specific secreted glycoprotein EP1
subtilisin-like protease SBT1.8
putative indole-3-acetaldehyde oxidase-like
putative phospholipid-transporting ATPase 8-like
putative cytochrome 83B1-like
DNA-directed RNA polymerase III subunit RPC2
Calcium-dependent protein kinase isoform 2
putative late blight resistance protein -like protein R1B-14 isoform 2
Aquaporin TIP1-2
putative pre-mRNA-processing factor 6-like
putative receptor-like protein 12-like
Cell division control protein 48 -like protein C
cation/H(+) antiporter 18-like
putative (+)-neomenthol dehydrogenase-like
Laccase-5
, chloroplastic-like
High-affinity nitrate transporter 2.1
putative multiple myeloma tumor-associated protein 2 -like protein
Eukaryotic translation initiation factor isoform 4G-1
putative cell number regulator 2-like
Phosphatidylinositol 4-kinase alpha 1
putative omega-6 fatty acid desaturase, endoplasmic reticulum isozyme 2-like
hypothetical protein FXO38_15768

e)

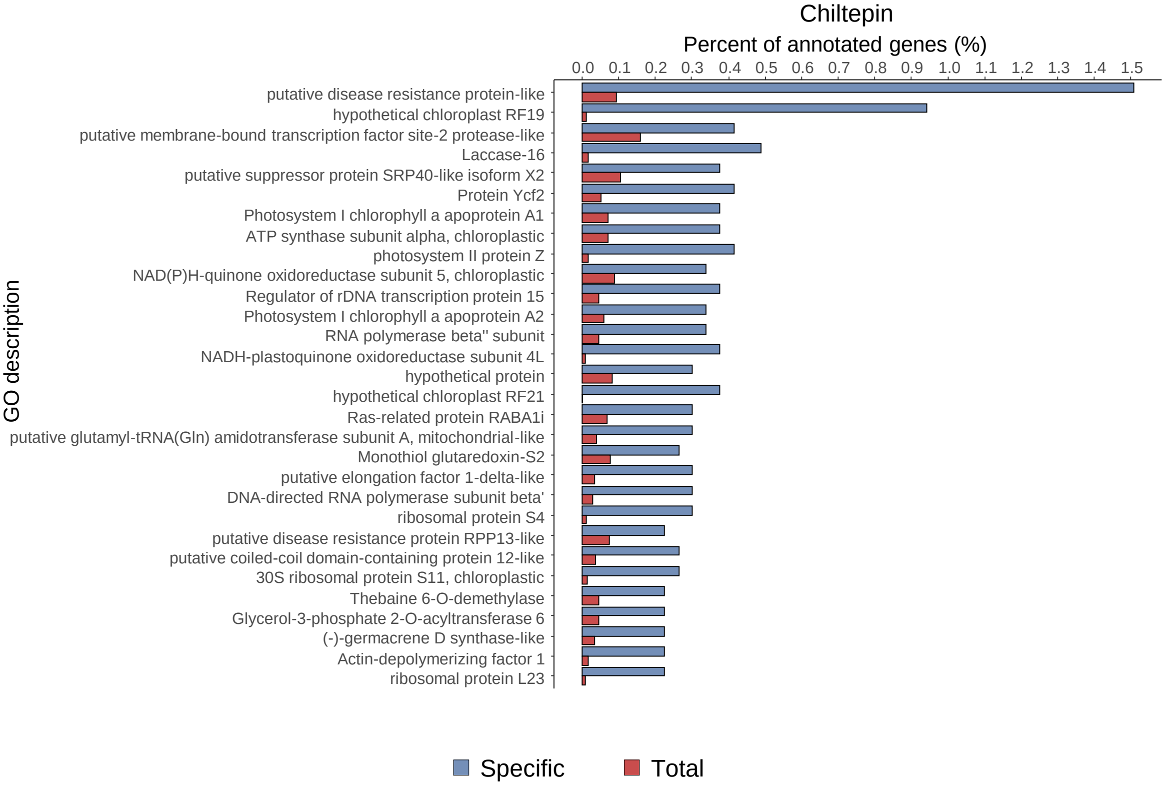

Supplementary Figure 5. Gene ontology enrichment analyses of unclustered genes in pepper accessions. The top 30 highest number of GO terms enriched in a) CM334, b) ECW, c) SF, d) Zunla-1, and e) Chiltepin specific genes were shown (P-value < 0.05).

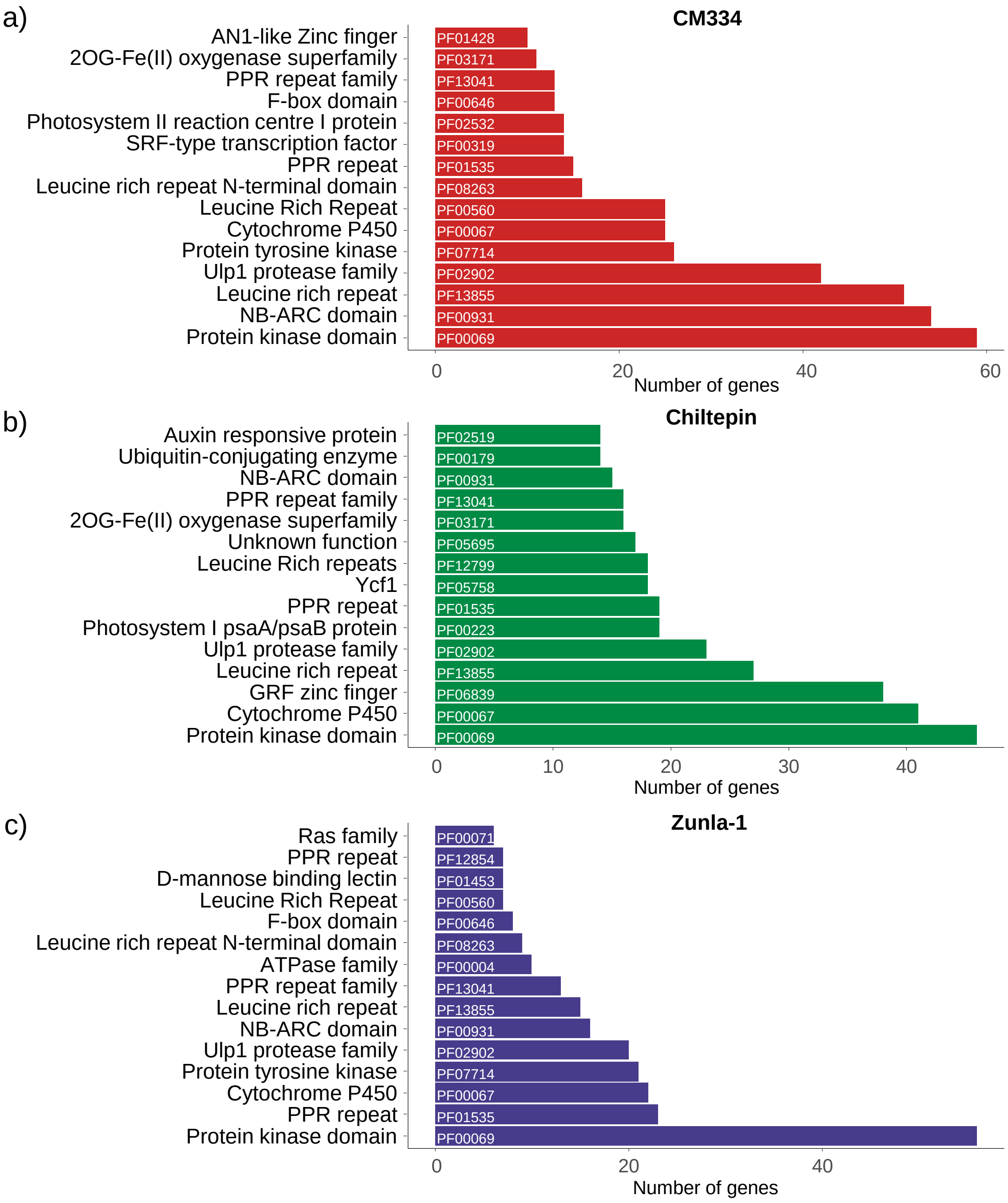

Supplementary Figure 6. The number of genes containing functional domains specific to CM334, Chiltepin, and Zunla-1. The top 15 highest number of functional domains not shared by other species were shown for a) CM334, b) Chiltepin, and c) Zunla-1.

a)

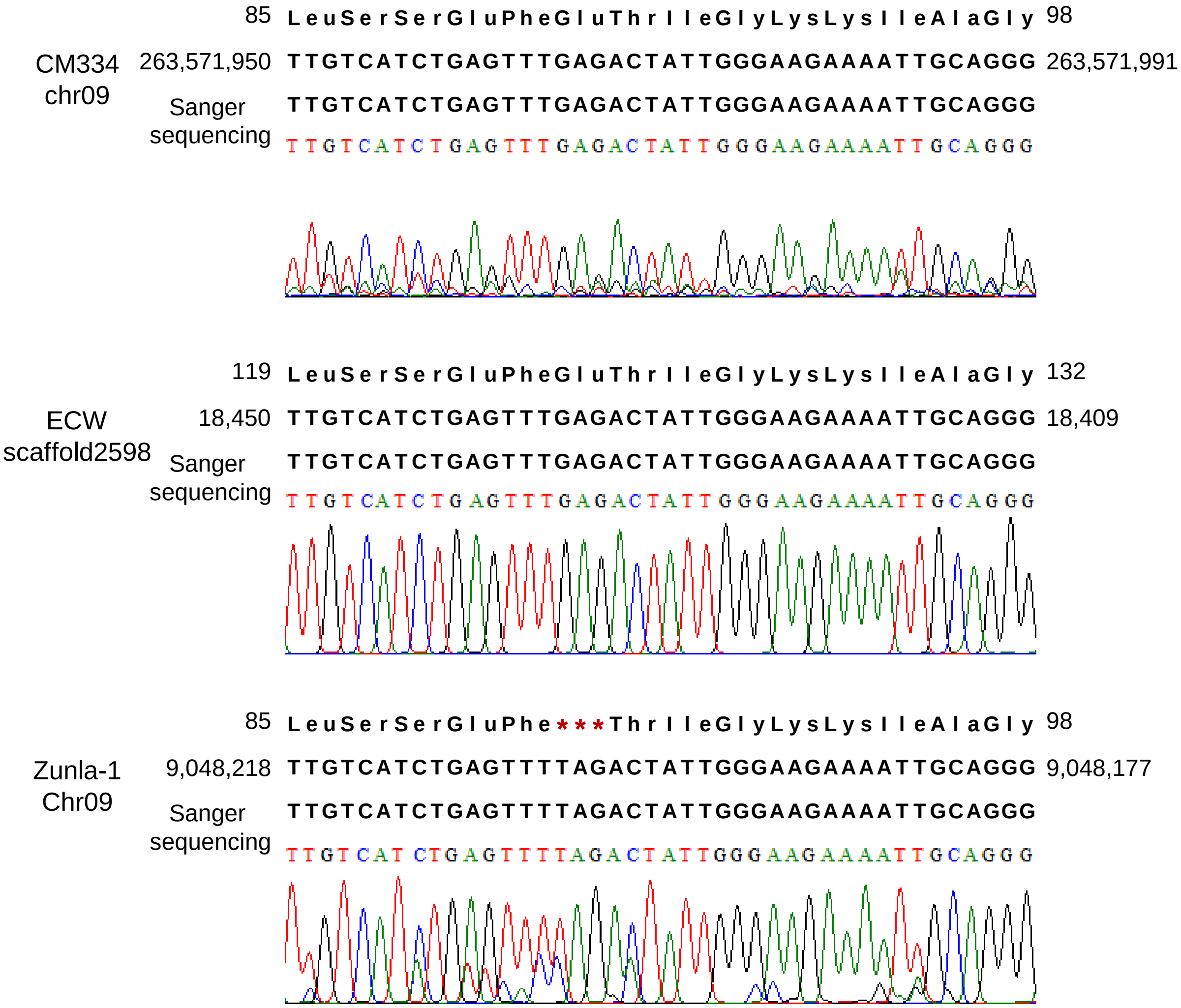
85 LeuSerSerGluPheGluThrIleGlyLysLysIleAlaGly 98
CM334 chr09
263,571,950 TTGTCATCTGAGTTTGAGACTATTGGGAAGAAAATTGCAGGG 263,571,991
Sanger sequencing
TTGTCATCTGAGTTTGAGACTATTGGGAAGAAAATTGCAGGG
TTGTCATCTGAGTTTGAGACTATTGGGAAGAAAATTGCAGGG
119 LeuSerSerGluPheGluThrIleGlyLysLysIleAlaGly 132
ECW scaffold2598
18,450 TTGTCATCTGAGTTTGAGACTATTGGGAAGAAAATTGCAGGG 18,409
Sanger sequencing
TTGTCATCTGAGTTTGAGACTATTGGGAAGAAAATTGCAGGG
TTGTCATCTGAGTTTGAGACTATTGGGAAGAAAATTGCAGGG
85 LeuSerSerGluPhe***ThrIleGlyLysLysIleAlaGly 98
Zunla-1 Chr09
9,048,218 TTGTCATCTGAGTTTTAGACTATTGGGAAGAAAATTGCAGGG 9,048,177
Sanger sequencing
TTGTCATCTGAGTTTTAGACTATTGGGAAGAAAATTGCAGGG
TTGTCATCTGAGTTTTAGACTATTGGGAAGAAAATTGCAGGG

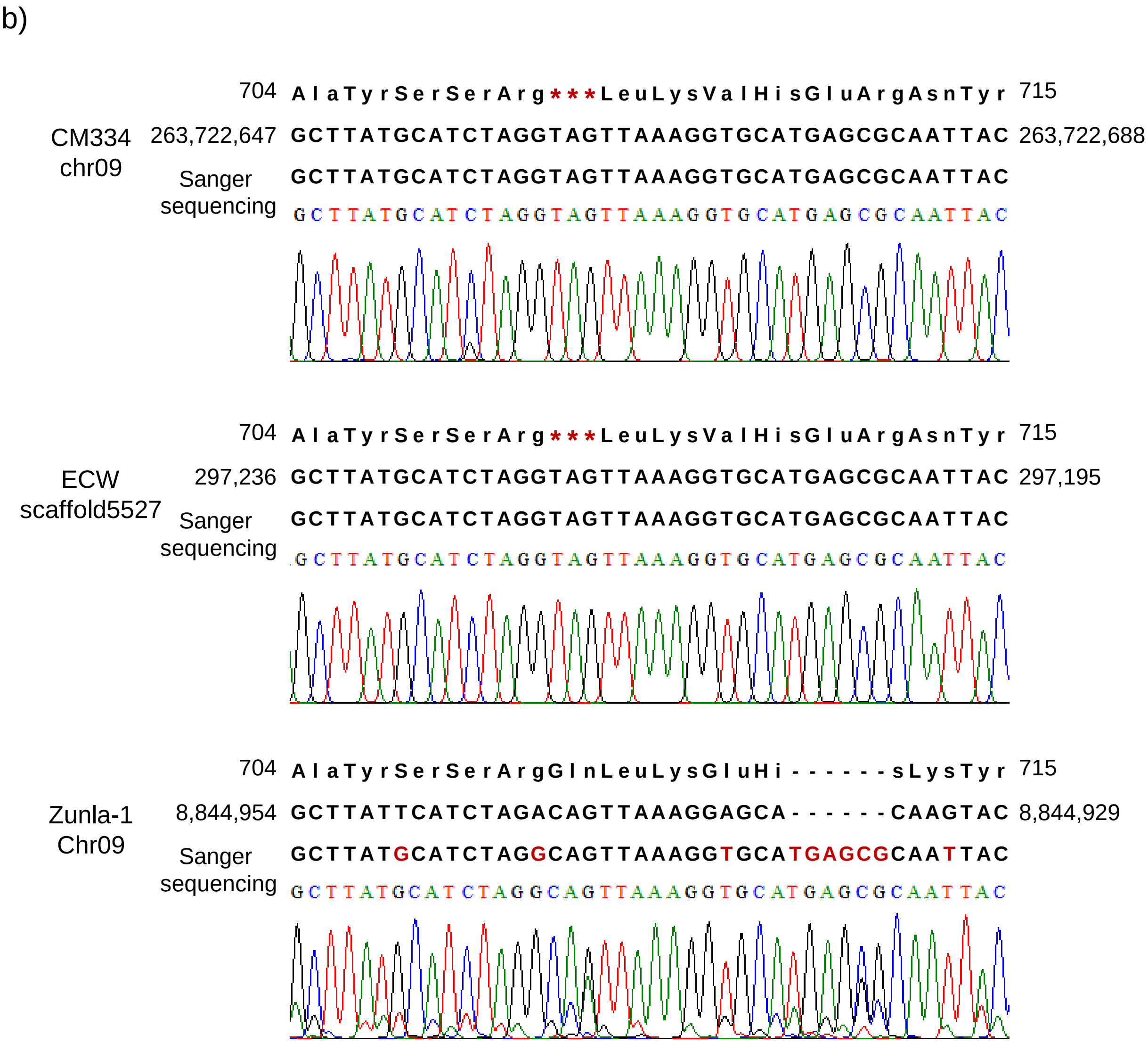

Supplementary Figure 7. PCR and sequencing validation for CNV of NLRs. The regions for early stop codons generated by small variation were amplified and sequenced in CM334, ECW, and Zunla-1. The early stop codon or mismatch between genome assembly and Sanger sequencing were highlighted in red. a) scaffold1090.36 in CM334, scaffold2598.10 in ECW, and corresponding genomic sequences in Zunla-1. b) Chr09.70 in Zunla-1 and corresponding genomic sequences in CM334 and ECW, respectively.
